# (*E*)-16-(4-Chloro­benzyl­idene)estrone

**DOI:** 10.1107/S1600536812050349

**Published:** 2012-12-19

**Authors:** J. Suresh, H. Thenmozhi, Veerappan Jeyachandran, R. Ranjith Kumar, P. L. Nilantha Lakshman

**Affiliations:** a Department of Physics, The Madura College, Madurai 625 011, India; b Department of Organic Chemistry, School of Chemistry, Madurai Kamaraj University, Madurai 625 021, India; c Department of Food Science and Technology, University of Ruhuna, Mapalana, Kamburupitiya 81100, Sri Lanka

## Abstract

In the title compound, C_25_H_25_ClO_2_, the *C* ring adopts a chair conformation, while the *B* ring approximates a half-chair conformation. The five-membered ring *D* has a twist con­form­ation on the C—C bond fused with the *C* ring. Aromatic rings *A* and *E* are not coplanar, as evidenced by the dihedral angle of 7.51 (1)°. In the crystal, O—H⋯O hydrogen bonds form a double chain along the *ab* plane inter­connected by C—H⋯O inter­actions.

## Related literature

For applications of steroids as radiodiagnostic compounds and drug delivery systems, see: Katzenellenbogen (1995 ▶); Silva *et al.* (2001 ▶); Wang *et al.* (2003 ▶). For related compounds, see: Cooper *et al.* (1969 ▶); Cody *et al.* (1971 ▶); Rajnikant *et al.* (2006 ▶); Gunasekaran *et al.* (2009 ▶). For conformational analysis of ring systems, see: Cremer & Pople (1975 ▶); Duax *et al.* (1976 ▶).
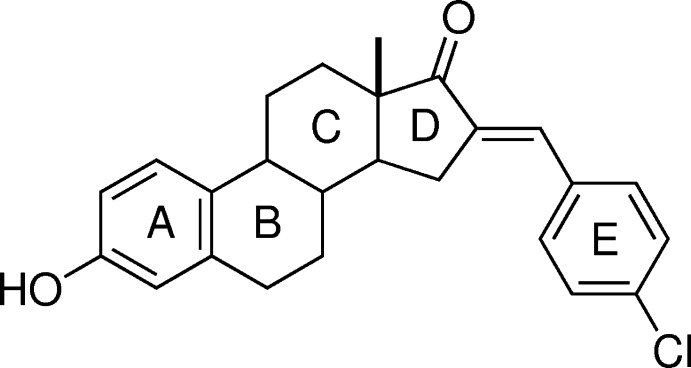


## Experimental

### 

#### Crystal data

C_25_H_25_ClO_2_
*M* *_r_* = 392.90
Orthorhombic, 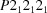

*a* = 6.3601 (3) Å
*b* = 11.2012 (6) Å
*c* = 28.3043 (14) Å
*V* = 2016.42 (18) Å^3^
*Z* = 4
Mo *K*α radiation
μ = 0.21 mm^−1^
*T* = 293 K
0.17 × 0.15 × 0.13 mm

#### Data collection

Bruker Kappa APEXII diffractometer
Absorption correction: multi-scan (*SADABS*; Sheldrick, 1996 ▶) *T*_min_ = 0.967, *T*_max_ = 0.974
23011 measured reflections
4819 independent reflections
3788 reflections with *I* > 2σ(*I*)
*R*_int_ = 0.034

#### Refinement

*R*[*F* ^2^ > 2σ(*F* ^2^)] = 0.043
*wR*(*F* ^2^) = 0.100
*S* = 1.02
4819 reflections
253 parameters
H-atom parameters constrained
Δρ_max_ = 0.22 e Å^−3^
Δρ_min_ = −0.26 e Å^−3^
Absolute structure: Flack (1983 ▶), 2037 Friedel pairs
Flack parameter: 0.06 (7)

### 

Data collection: *APEX2* (Bruker, 2004 ▶); cell refinement: *SAINT* (Bruker, 2004 ▶); data reduction: *SAINT*; program(s) used to solve structure: *SHELXS97* (Sheldrick, 2008 ▶); program(s) used to refine structure: *SHELXL97* (Sheldrick, 2008 ▶); molecular graphics: *PLATON* (Spek, 2009 ▶); software used to prepare material for publication: *SHELXL97*.

## Supplementary Material

Crystal structure: contains datablock(s) global, I. DOI: 10.1107/S1600536812050349/bh2467sup1.cif

Structure factors: contains datablock(s) I. DOI: 10.1107/S1600536812050349/bh2467Isup2.hkl

Additional supplementary materials:  crystallographic information; 3D view; checkCIF report

## Figures and Tables

**Table 1:** Hydrogen-bond geometry (Å, °)

*D*—H⋯*A*	*D*—H	H⋯*A*	*D*⋯*A*	*D*—H⋯*A*
O1—H1⋯O2^i^	0.82	2.03	2.762 (3)	148
C14*A*—H14*A*⋯O1^ii^	0.96	2.55	3.454 (3)	157

Symmetry codes: (i) 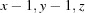; (ii) 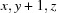.

**Fig. 1.**
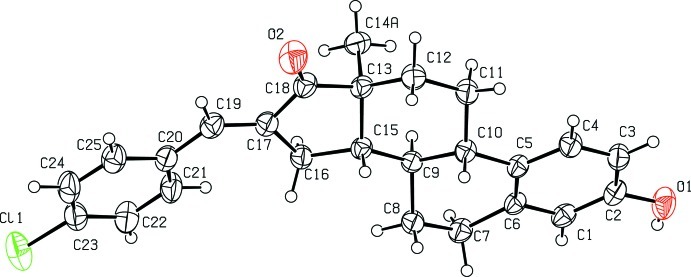
The molecular structure of the title compound, showing 40% probability displacement ellipsoids.

**Fig. 2.**
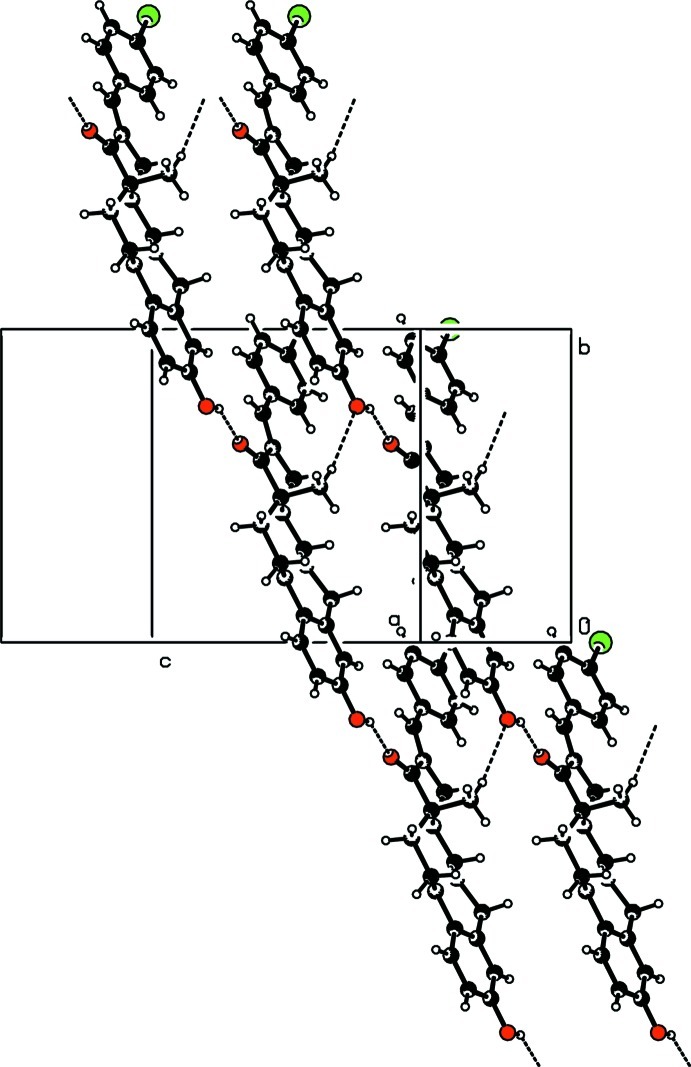
Partial packing diagram showing double chains formation.

## supplementary crystallographic information

### Comment

Synthetic steroids have been proposed as radiodiagnostic compounds (Katzenellenbogen, 1995; Silva *et al.*, 2001; Wang *et al.*, 2003), as well as potential drug delivery systems targeting oestrogen receptor positive breast cancer and other diseases associated with the oestrogen receptor ERα. The medicinal importance of the compound in conjunction with our research interests prompted us to synthesize and report the X-ray structure of the title compound.

In the title compound (Fig. 1), ring *C*, with *trans* fusion to rings *B* and *D*, is fixed in a chair conformation, and the ring *D* adopts a slightly twisted envelope conformation, as characterized by the puckering parameters, *q*_2_ = 0.422 (3) Å and φ = 227.8 (2)° (Cremer & Pople, 1975). The steric repulsive hindrance is reduced by a twisting about the C5—C10 bond (Cooper *et al.*, 1969), leading to a slightly deformed half-chair conformation for ring *B* (Cody *et al.*, 1971), which is supported by the puckering parameters *q*_2_ = 0.528 (3) Å, φ = 128.5 (2) ° and θ = 227.8 (2)°. Ring *A* displays typical characteristic aromaticity, with delocalization of π electrons producing an average C≐C bond length of 1.381 (3) Å (Duax *et al.*, 1976). Additionally, the C4≐C5≐C6 bond angle is reduced to a value of 117.27 (16)°. These facts are due to a strong interaction of the aromatic atom H4 of ring *A* and equatorial atom H11 atom on ring *C*, indicated by an interatomic H···H separation of 2.118 (5) Å. The aromatic rings *A* and *E* are not coplanar, as evidenced by the dihedral angle of 7.51 (1)° between them. All these features are consistent with previously reported structures for similar compounds (Rajnikant *et al.*, 2006; Gunasekaran *et al.*, 2009).

The crystal structure features intermolecular C—H···O and O—H···O interactions in addition to a weak C—H···O intramolecular interaction. The O—H···O interactions form a double chain interconnected by the C—H···O interactions (Fig. 2).

### Experimental

A mixture of oestrone (1 mmol), 4-chloro benzaldehyde (1 mmol), potassium hydroxide (5 ml, 20%) in ethanol (5 ml) was refluxed on an oil bath with stirring at 120 °C for 5 h. After completion of the reaction, as indicated by TLC, the reaction mixture was poured into ice-water (50 ml). The product was filtered and washed with water (100 ml) to obtain the title compound, which was dried under vacuum. The compound was further recrystallized from ethanol to obtain suitable crystals for X-ray analysis. Melting point: 185–186 °C, Yield: 82%.

### Refinement

For the title compound, the absolute configuration expected from the starting reagents was confirmed by the refinement of the Flack parameter (2037 Friedel pairs; Flack, 1983). H atoms were placed at calculated positions and allowed to ride on their carrier atoms with C—H = 0.93–0.98 Å, O—H = 0.82 Å, *U*_iso_(H) =1.2*U*_eq_(carrier C) for CH_2_ and CH groups, *U*_iso_(H)=1.5*U*_eq_(C14A) for the CH_3_ group, and *U*_iso_(H1)=1.5*U*_eq_(O1).

### Crystal data

**Table:**

C_25_H_25_ClO_2_	*D*_x_ = 1.294 Mg m^−^^3^
*M**_r_* = 392.90	Melting point: 458 K
Orthorhombic, *P*2_1_2_1_2_1_	Mo *K*α radiation, λ = 0.71073 Å
Hall symbol: P 2ac 2ab	Cell parameters from 2000 reflections
*a* = 6.3601 (3) Å	θ = 2–31°
*b* = 11.2012 (6) Å	µ = 0.21 mm^−^^1^
*c* = 28.3043 (14) Å	*T* = 293 K
*V* = 2016.42 (18) Å^3^	Block, colourless
*Z* = 4	0.17 × 0.15 × 0.13 mm
*F*(000) = 832	

### Data collection

**Table:**

Bruker Kappa APEXII diffractometer	4819 independent reflections
Radiation source: fine-focus sealed tube	3788 reflections with *I* > 2σ(*I*)
Graphite monochromator	*R*_int_ = 0.034
Detector resolution: 0 pixels mm^-1^	θ_max_ = 27.9°, θ_min_ = 2.3°
ω and φ scans	*h* = −8→7
Absorption correction: multi-scan (*SADABS*; Sheldrick, 1996)	*k* = −14→14
*T*_min_ = 0.967, *T*_max_ = 0.974	*l* = −37→36
23011 measured reflections	

### Refinement

**Table:**

Refinement on *F*^2^	Secondary atom site location: difference Fourier map
Least-squares matrix: full	Hydrogen site location: inferred from neighbouring sites
*R*[*F*^2^ > 2σ(*F*^2^)] = 0.043	H-atom parameters constrained
*wR*(*F*^2^) = 0.100	*w* = 1/[σ^2^(*F*_o_^2^) + (0.0435*P*)^2^ + 0.2966*P*] where *P* = (*F*_o_^2^ + 2*F*_c_^2^)/3
*S* = 1.02	(Δ/σ)_max_ = 0.001
4819 reflections	Δρ_max_ = 0.22 e Å^−^^3^
253 parameters	Δρ_min_ = −0.26 e Å^−^^3^
0 restraints	Absolute structure: Flack (1983), 2037 Friedel pairs
0 constraints	Flack parameter: 0.06 (7)
Primary atom site location: structure-invariant direct methods	

### Fractional atomic coordinates and isotropic or equivalent isotropic displacement parameters (Å^2^)

**Table:**

	*x*	*y*	*z*	*U*_iso_*/*U*_eq_	
C1	0.2517 (3)	−0.05590 (16)	0.09206 (6)	0.0420 (4)	
H1A	0.1200	−0.0754	0.1041	0.050*	
C2	0.3552 (3)	−0.13702 (16)	0.06397 (7)	0.0448 (4)	
C3	0.5521 (3)	−0.10893 (17)	0.04649 (7)	0.0478 (5)	
H3	0.6251	−0.1634	0.0279	0.057*	
C4	0.6387 (3)	0.00039 (17)	0.05695 (7)	0.0451 (4)	
H4	0.7713	0.0184	0.0451	0.054*	
C5	0.5365 (3)	0.08533 (15)	0.08452 (6)	0.0369 (4)	
C6	0.3391 (3)	0.05456 (16)	0.10293 (6)	0.0367 (4)	
C7	0.2169 (3)	0.13852 (16)	0.13436 (7)	0.0452 (5)	
H7A	0.1693	0.0949	0.1620	0.054*	
H7B	0.0932	0.1657	0.1174	0.054*	
C8	0.3413 (3)	0.24637 (17)	0.15048 (7)	0.0456 (5)	
H8A	0.4355	0.2234	0.1759	0.055*	
H8B	0.2459	0.3067	0.1625	0.055*	
C9	0.4680 (3)	0.29775 (15)	0.10975 (6)	0.0358 (4)	
H9	0.3731	0.3114	0.0830	0.043*	
C10	0.6358 (3)	0.20596 (16)	0.09476 (6)	0.0367 (4)	
H10	0.7275	0.1944	0.1222	0.044*	
C11	0.7758 (3)	0.25270 (16)	0.05509 (7)	0.0461 (5)	
H11A	0.6930	0.2589	0.0264	0.055*	
H11B	0.8873	0.1954	0.0493	0.055*	
C12	0.8747 (3)	0.37515 (17)	0.06580 (7)	0.0486 (5)	
H12A	0.9754	0.3673	0.0914	0.058*	
H12B	0.9489	0.4040	0.0381	0.058*	
C13	0.7053 (3)	0.46360 (16)	0.07958 (6)	0.0394 (4)	
C14A	0.5673 (3)	0.49699 (18)	0.03654 (7)	0.0533 (5)	
H14A	0.4614	0.5531	0.0461	0.080*	
H14B	0.5012	0.4264	0.0243	0.080*	
H14C	0.6537	0.5322	0.0125	0.080*	
C15	0.5779 (3)	0.41378 (15)	0.12129 (6)	0.0362 (4)	
H15	0.6805	0.3949	0.1460	0.043*	
C16	0.4532 (3)	0.52206 (15)	0.13940 (7)	0.0420 (4)	
H16A	0.3278	0.5355	0.1206	0.050*	
H16B	0.4135	0.5122	0.1723	0.050*	
C17	0.6101 (3)	0.62256 (17)	0.13336 (7)	0.0433 (4)	
C18	0.7770 (3)	0.58149 (17)	0.10050 (7)	0.0449 (4)	
C19	0.6214 (3)	0.73160 (17)	0.15245 (7)	0.0464 (5)	
H19	0.7401	0.7754	0.1442	0.056*	
C20	0.4751 (3)	0.79255 (16)	0.18433 (7)	0.0459 (4)	
C21	0.2732 (4)	0.75169 (18)	0.19375 (8)	0.0574 (6)	
H21	0.2274	0.6811	0.1798	0.069*	
C22	0.1395 (4)	0.8131 (2)	0.22317 (8)	0.0610 (6)	
H22	0.0065	0.7831	0.2299	0.073*	
C23	0.2042 (4)	0.91873 (19)	0.24246 (7)	0.0544 (5)	
C24	0.4006 (4)	0.9632 (2)	0.23352 (8)	0.0598 (6)	
H24	0.4434	1.0350	0.2469	0.072*	
C25	0.5342 (4)	0.90013 (18)	0.20449 (7)	0.0538 (5)	
H25	0.6674	0.9305	0.1983	0.065*	
O1	0.2750 (2)	−0.24606 (12)	0.05227 (6)	0.0622 (4)	
H1	0.1584	−0.2538	0.0642	0.093*	
O2	0.9391 (2)	0.63375 (13)	0.09181 (6)	0.0698 (5)	
Cl1	0.03190 (11)	0.99936 (6)	0.27780 (3)	0.0845 (2)	

### Atomic displacement parameters (Å^2^)

**Table:**

	*U*^11^	*U*^22^	*U*^33^	*U*^12^	*U*^13^	*U*^23^
C1	0.0336 (9)	0.0398 (10)	0.0525 (11)	−0.0008 (8)	0.0016 (8)	0.0097 (9)
C2	0.0501 (11)	0.0320 (10)	0.0524 (11)	−0.0018 (8)	−0.0056 (9)	0.0039 (8)
C3	0.0495 (11)	0.0361 (10)	0.0577 (11)	0.0013 (9)	0.0092 (10)	−0.0032 (9)
C4	0.0377 (9)	0.0409 (11)	0.0568 (11)	0.0017 (8)	0.0105 (9)	−0.0006 (9)
C5	0.0341 (8)	0.0333 (9)	0.0431 (9)	0.0020 (8)	0.0006 (7)	0.0032 (7)
C6	0.0339 (8)	0.0325 (9)	0.0438 (9)	0.0038 (7)	0.0002 (7)	0.0062 (8)
C7	0.0378 (9)	0.0400 (10)	0.0580 (11)	−0.0005 (8)	0.0134 (9)	0.0061 (9)
C8	0.0465 (10)	0.0391 (11)	0.0512 (11)	0.0010 (9)	0.0156 (9)	−0.0010 (9)
C9	0.0342 (8)	0.0346 (9)	0.0386 (9)	0.0004 (7)	0.0058 (7)	0.0005 (7)
C10	0.0312 (8)	0.0350 (9)	0.0439 (10)	0.0017 (7)	0.0010 (8)	0.0002 (8)
C11	0.0417 (10)	0.0403 (10)	0.0564 (11)	−0.0024 (9)	0.0148 (9)	−0.0033 (9)
C12	0.0417 (10)	0.0448 (11)	0.0592 (12)	−0.0069 (9)	0.0138 (9)	−0.0033 (9)
C13	0.0384 (9)	0.0354 (10)	0.0445 (10)	−0.0052 (8)	0.0049 (8)	0.0023 (8)
C14A	0.0700 (13)	0.0475 (12)	0.0425 (10)	−0.0061 (11)	−0.0005 (9)	0.0057 (9)
C15	0.0350 (8)	0.0336 (9)	0.0399 (9)	−0.0022 (7)	0.0014 (7)	0.0010 (7)
C16	0.0439 (9)	0.0369 (10)	0.0452 (10)	−0.0026 (8)	0.0046 (8)	−0.0027 (8)
C17	0.0461 (10)	0.0369 (10)	0.0469 (10)	−0.0015 (8)	−0.0024 (8)	0.0003 (8)
C18	0.0425 (10)	0.0384 (10)	0.0538 (11)	−0.0061 (9)	0.0019 (9)	0.0023 (9)
C19	0.0480 (11)	0.0397 (11)	0.0515 (11)	−0.0076 (9)	−0.0042 (9)	0.0014 (9)
C20	0.0550 (12)	0.0349 (10)	0.0478 (10)	0.0000 (9)	−0.0085 (9)	−0.0002 (8)
C21	0.0620 (13)	0.0367 (11)	0.0733 (14)	−0.0061 (10)	0.0060 (11)	−0.0110 (10)
C22	0.0599 (13)	0.0450 (12)	0.0783 (15)	0.0008 (10)	0.0035 (12)	−0.0082 (11)
C23	0.0647 (13)	0.0475 (12)	0.0509 (12)	0.0134 (11)	−0.0067 (10)	−0.0081 (10)
C24	0.0730 (15)	0.0440 (12)	0.0625 (13)	0.0030 (11)	−0.0198 (11)	−0.0163 (10)
C25	0.0563 (12)	0.0451 (11)	0.0601 (12)	−0.0018 (10)	−0.0122 (10)	−0.0041 (10)
O1	0.0611 (9)	0.0404 (8)	0.0852 (10)	−0.0128 (7)	0.0066 (8)	−0.0107 (7)
O2	0.0550 (9)	0.0561 (9)	0.0982 (12)	−0.0228 (8)	0.0191 (9)	−0.0089 (9)
Cl1	0.0868 (4)	0.0770 (5)	0.0897 (4)	0.0234 (4)	−0.0009 (4)	−0.0325 (3)

### Geometric parameters (Å, º)

**Table:**

C1—C2	1.375 (3)	C13—C18	1.517 (3)
C1—C6	1.391 (3)	C13—C15	1.537 (2)
C1—H1A	0.9300	C13—C14A	1.547 (3)
C2—O1	1.364 (2)	C14A—H14A	0.9600
C2—C3	1.382 (3)	C14A—H14B	0.9600
C3—C4	1.375 (3)	C14A—H14C	0.9600
C3—H3	0.9300	C15—C16	1.537 (2)
C4—C5	1.391 (2)	C15—H15	0.9800
C4—H4	0.9300	C16—C17	1.514 (3)
C5—C6	1.403 (2)	C16—H16A	0.9700
C5—C10	1.519 (3)	C16—H16B	0.9700
C6—C7	1.510 (3)	C17—C19	1.337 (3)
C7—C8	1.515 (3)	C17—C18	1.484 (3)
C7—H7A	0.9700	C18—O2	1.211 (2)
C7—H7B	0.9700	C19—C20	1.465 (3)
C8—C9	1.520 (2)	C19—H19	0.9300
C8—H8A	0.9700	C20—C25	1.385 (3)
C8—H8B	0.9700	C20—C21	1.389 (3)
C9—C15	1.511 (2)	C21—C22	1.375 (3)
C9—C10	1.542 (2)	C21—H21	0.9300
C9—H9	0.9800	C22—C23	1.367 (3)
C10—C11	1.526 (2)	C22—H22	0.9300
C10—H10	0.9800	C23—C24	1.368 (3)
C11—C12	1.539 (3)	C23—Cl1	1.737 (2)
C11—H11A	0.9700	C24—C25	1.376 (3)
C11—H11B	0.9700	C24—H24	0.9300
C12—C13	1.515 (3)	C25—H25	0.9300
C12—H12A	0.9700	O1—H1	0.8200
C12—H12B	0.9700		
			
C2—C1—C6	121.61 (16)	H12A—C12—H12B	108.2
C2—C1—H1A	119.2	C12—C13—C18	117.13 (15)
C6—C1—H1A	119.2	C12—C13—C15	109.59 (14)
O1—C2—C1	123.56 (18)	C18—C13—C15	100.06 (14)
O1—C2—C3	117.09 (18)	C12—C13—C14A	111.03 (16)
C1—C2—C3	119.35 (17)	C18—C13—C14A	105.50 (15)
C4—C3—C2	119.25 (18)	C15—C13—C14A	113.17 (15)
C4—C3—H3	120.4	C13—C14A—H14A	109.5
C2—C3—H3	120.4	C13—C14A—H14B	109.5
C3—C4—C5	122.86 (17)	H14A—C14A—H14B	109.5
C3—C4—H4	118.6	C13—C14A—H14C	109.5
C5—C4—H4	118.6	H14A—C14A—H14C	109.5
C4—C5—C6	117.27 (16)	H14B—C14A—H14C	109.5
C4—C5—C10	121.40 (16)	C9—C15—C13	112.96 (14)
C6—C5—C10	121.32 (16)	C9—C15—C16	120.79 (14)
C1—C6—C5	119.63 (16)	C13—C15—C16	103.99 (13)
C1—C6—C7	118.60 (16)	C9—C15—H15	106.0
C5—C6—C7	121.77 (16)	C13—C15—H15	106.0
C6—C7—C8	113.92 (15)	C16—C15—H15	106.0
C6—C7—H7A	108.8	C17—C16—C15	102.05 (14)
C8—C7—H7A	108.8	C17—C16—H16A	111.4
C6—C7—H7B	108.8	C15—C16—H16A	111.4
C8—C7—H7B	108.8	C17—C16—H16B	111.4
H7A—C7—H7B	107.7	C15—C16—H16B	111.4
C7—C8—C9	110.52 (15)	H16A—C16—H16B	109.2
C7—C8—H8A	109.5	C19—C17—C18	119.86 (18)
C9—C8—H8A	109.5	C19—C17—C16	131.97 (18)
C7—C8—H8B	109.5	C18—C17—C16	108.15 (15)
C9—C8—H8B	109.5	O2—C18—C17	125.89 (18)
H8A—C8—H8B	108.1	O2—C18—C13	126.68 (18)
C15—C9—C8	114.01 (14)	C17—C18—C13	107.43 (15)
C15—C9—C10	108.23 (14)	C17—C19—C20	129.83 (18)
C8—C9—C10	108.86 (14)	C17—C19—H19	115.1
C15—C9—H9	108.5	C20—C19—H19	115.1
C8—C9—H9	108.5	C25—C20—C21	117.27 (19)
C10—C9—H9	108.5	C25—C20—C19	119.16 (19)
C5—C10—C11	114.03 (15)	C21—C20—C19	123.48 (17)
C5—C10—C9	110.97 (14)	C22—C21—C20	121.56 (19)
C11—C10—C9	112.19 (14)	C22—C21—H21	119.2
C5—C10—H10	106.4	C20—C21—H21	119.2
C11—C10—H10	106.4	C23—C22—C21	119.3 (2)
C9—C10—H10	106.4	C23—C22—H22	120.4
C10—C11—C12	113.53 (15)	C21—C22—H22	120.4
C10—C11—H11A	108.9	C22—C23—C24	121.1 (2)
C12—C11—H11A	108.9	C22—C23—Cl1	119.36 (18)
C10—C11—H11B	108.9	C24—C23—Cl1	119.57 (17)
C12—C11—H11B	108.9	C23—C24—C25	119.16 (19)
H11A—C11—H11B	107.7	C23—C24—H24	120.4
C13—C12—C11	110.05 (15)	C25—C24—H24	120.4
C13—C12—H12A	109.7	C24—C25—C20	121.7 (2)
C11—C12—H12A	109.7	C24—C25—H25	119.2
C13—C12—H12B	109.7	C20—C25—H25	119.2
C11—C12—H12B	109.7	C2—O1—H1	109.5
			
C6—C1—C2—O1	−179.72 (17)	C10—C9—C15—C16	−177.53 (15)
C6—C1—C2—C3	0.8 (3)	C12—C13—C15—C9	−61.04 (19)
O1—C2—C3—C4	179.40 (17)	C18—C13—C15—C9	175.26 (14)
C1—C2—C3—C4	−1.1 (3)	C14A—C13—C15—C9	63.48 (19)
C2—C3—C4—C5	−0.1 (3)	C12—C13—C15—C16	166.24 (15)
C3—C4—C5—C6	1.6 (3)	C18—C13—C15—C16	42.54 (17)
C3—C4—C5—C10	−179.11 (17)	C14A—C13—C15—C16	−69.24 (18)
C2—C1—C6—C5	0.7 (3)	C9—C15—C16—C17	−165.37 (15)
C2—C1—C6—C7	−179.44 (17)	C13—C15—C16—C17	−37.32 (17)
C4—C5—C6—C1	−1.8 (2)	C15—C16—C17—C19	−161.1 (2)
C10—C5—C6—C1	178.84 (16)	C15—C16—C17—C18	17.24 (19)
C4—C5—C6—C7	178.34 (17)	C19—C17—C18—O2	7.3 (3)
C10—C5—C6—C7	−1.0 (3)	C16—C17—C18—O2	−171.3 (2)
C1—C6—C7—C8	170.23 (16)	C19—C17—C18—C13	−172.01 (17)
C5—C6—C7—C8	−9.9 (2)	C16—C17—C18—C13	9.4 (2)
C6—C7—C8—C9	43.0 (2)	C12—C13—C18—O2	30.6 (3)
C7—C8—C9—C15	173.49 (15)	C15—C13—C18—O2	148.9 (2)
C7—C8—C9—C10	−65.58 (19)	C14A—C13—C18—O2	−93.5 (2)
C4—C5—C10—C11	31.8 (2)	C12—C13—C18—C17	−150.10 (16)
C6—C5—C10—C11	−148.94 (16)	C15—C13—C18—C17	−31.83 (18)
C4—C5—C10—C9	159.62 (16)	C14A—C13—C18—C17	85.80 (17)
C6—C5—C10—C9	−21.1 (2)	C18—C17—C19—C20	178.14 (18)
C15—C9—C10—C5	177.72 (14)	C16—C17—C19—C20	−3.7 (4)
C8—C9—C10—C5	53.30 (19)	C17—C19—C20—C25	171.5 (2)
C15—C9—C10—C11	−53.43 (19)	C17—C19—C20—C21	−12.1 (3)
C8—C9—C10—C11	−177.85 (15)	C25—C20—C21—C22	−2.2 (3)
C5—C10—C11—C12	179.77 (15)	C19—C20—C21—C22	−178.62 (19)
C9—C10—C11—C12	52.5 (2)	C20—C21—C22—C23	2.1 (3)
C10—C11—C12—C13	−53.1 (2)	C21—C22—C23—C24	−1.0 (3)
C11—C12—C13—C18	168.38 (16)	C21—C22—C23—Cl1	177.52 (17)
C11—C12—C13—C15	55.4 (2)	C22—C23—C24—C25	0.2 (3)
C11—C12—C13—C14A	−70.4 (2)	Cl1—C23—C24—C25	−178.34 (16)
C8—C9—C15—C13	179.84 (15)	C23—C24—C25—C20	−0.4 (3)
C10—C9—C15—C13	58.55 (18)	C21—C20—C25—C24	1.4 (3)
C8—C9—C15—C16	−56.2 (2)	C19—C20—C25—C24	177.93 (19)

### Hydrogen-bond geometry (Å, º)

**Table:**

*D*—H···*A*	*D*—H	H···*A*	*D*···*A*	*D*—H···*A*
O1—H1···O2^i^	0.82	2.03	2.762 (3)	148
C14*A*—H14*A*···O1^ii^	0.96	2.55	3.454 (3)	157

Symmetry codes: (i) *x*−1, *y*−1, *z*; (ii) *x*, *y*+1, *z*.
